# A Complex and Evolutive Character: Two Face Aspects of ECM in Tumor Progression

**DOI:** 10.3389/fonc.2020.01620

**Published:** 2020-08-28

**Authors:** Margaux Sala, Manon Ros, Frédéric Saltel

**Affiliations:** Univ. Bordeaux, INSERM, BaRITOn, U1053, Bordeaux, France

**Keywords:** extracellular matrix, invasion, invadosome, CAFs, matrix protective role, matrix promoting role

## Abstract

Tumor microenvironment, including extracellular matrix (ECM) and stromal cells, is a key player during tumor development, from initiation, growth and progression to metastasis. During all of these steps, remodeling of matrix components occurs, changing its biochemical and physical properties. The global and basic cancer ECM model is that tumors are surrounded by activated stromal cells, that remodel physiological ECM to evolve into a stiffer and more crosslinked ECM than in normal conditions, thereby increasing invasive capacities of cancer cells. In this review, we show that this too simple model does not consider the complexity, specificity and heterogeneity of each organ and tumor. First, we describe the general ECM in context of cancer. Then, we go through five invasive and most frequent cancers from different origins (breast, liver, pancreas, colon, and skin), and show that each cancer has its own specific matrix, with different stromal cells, ECM components, biochemical properties and activated signaling pathways. Furthermore, in these five cancers, we describe the dual role of tumor ECM: as a protective barrier against tumor cell proliferation and invasion, and as a major player in tumor progression. Indeed, crosstalk between tumor and stromal cells induce changes in matrix organization by remodeling ECM through invadosome formation in order to degrade it, promoting tumor progression and cell invasion. To sum up, in this review, we highlight the specificities of matrix composition in five cancers and the necessity not to consider the ECM as one general and simple entity, but one complex, dynamic and specific entity for each cancer type and subtype.

## Introduction

In 2012, Hanahan et al. reviewed the hallmarks of cancer by including the tumor microenvironment (1). This concept postulates that cancer cells are not able to promote the disease alone but they could recruit and modulate resident and normal cell types in order to establish cooperation to promote tumor progression (2). The tumor microenvironment is a complex and dynamic network composed of cancer cells, stromal tissue (stromal cells such as fibroblasts, macrophages, immune cells, cytokines, and vascular tissue), as well as the extracellular matrix (ECM) (3). ECM plays key roles during tumor development, from initiation, growth and progression to metastasis (2). Remodeling of matrix components occurs during all of these steps. The role of the ECM in this journey is still not emphasized enough with the exception of some studies (4–7).

The ECM is the acellular component, secreted by the cells, that forms a tissue. It has a supporting role for normal cells, as well as a role in maintaining tissue homeostasis. In addition, the ECM is also involved in the establishment, separation and maintenance of differentiated tissues and organs (8). Structurally, ECM proteins notably form the basement membrane (BM), which separate the epithelium or endothelium from the stroma and the interstitial matrix involved in tissue resistance (9). The ECM composition can be very different according to the tissue, due to the wide variety of proteins involved in its composition. ECM is composed of hydrated gel-forming macromolecules [hyaluronic acid (HA), proteoglycans], fibrillar proteins (collagen) and structural proteins (elastin and fibronectin). These macromolecules can assemble together to form three dimensional supramolecular structures with distinct biochemical and biophysical properties (10). Cells can interact with the ECM through expression of receptors at their cell surface, in order to maintain physiological signaling such as homeostasis, adhesion and migration.

In addition to its structural role, the ECM has a reservoir role for bioactive molecules such as cytokines and growth factors. ECM is then involved in cell growth, proliferation, survival, differentiation, migration and invasion (9). ECM is a dynamic environment which is constantly remodeled to adapt and maintain tissue homeostasis (11). This remodeling process is deregulated during cancer, with abnormal ECM deposition and stiffness, leading to tumor progression (12). In order to sense, remodel and degrade the ECM, matrix receptors such as CD44, integrins or discoidin domain receptors contribute to formation of invasive structures called invadosomes (or invadopodia), allowing invasion of cancer cells and metastasis formation (13, 14). However, this classical model of ECM remodeling with increased crosslinking, stiffness and tumor-promoting signaling pathway activation does not apply to all stages of all cancers. This model does not consider ECM heterogeneity, complexity and specificity of each organ and each tumor. Indeed, each organ possesses its own ECM with unique architecture, composition and biological and physical properties associated with organ specific roles (9). Most of the studies still consider the model of tumor ECM as one entity without discriminating each cancer type. Indeed, studies are usually performed on a 2-dimensional (2D) matrix made of only one matrix element, vitronectin, laminin and quite often, collagen I. Moreover, those matrix elements are not in their physiological organization. For example, type I collagen is used as monomers and not in its physiological triple helix form, which does not reflect the *in vivo* ECM. It will be interesting to study and compare all tumors and associated extracellular matrices, in order to create more complex and relevant ECM networks to work with.

The ECM is also the interface between tumor cells and normal tissues. This interface evolves over time, in parallel with the tumor. Initially, the ECM forms a physical barrier, preventing the proliferation and invasion of tumor cells and then, plays a protective role (15). We could hypothesize that stresses, such as hypoxia, oxidative or metabolic stresses, proliferation of tumor cells or ECM accumulation could lower protective nature of the matrix and favor tumor progression. Consequently, dialogue between tumor cells and surrounding ECM is a key element in the tumor progression process by promoting tumor cell invasion (9, 12). So far, there have been no studies on the ECM’s protective barrier role, and as such, this molecular mechanism needs further investigations. The basic scheme of tumor associated matrix is that ECM remodeling process is abnormally deregulated during cancer, with an increase in ECM deposition and degradation, promoting tumor invasion.

In this review, we describe the main molecular components of the ECM and associated biomechanical properties. We describe the ECM composition and its role in five cancers (breast, liver, pancreas, colon cancer, and melanoma), highlight their similarities and differences, show that each cancer possesses its own specific matrix associated with physical and biochemical properties. Furthermore, in these five cancers, we evaluate the protective and the pro-invasive role of the ECM.

To sum up, in order to go beyond the classical and reducing scheme of the tumor-associated ECM, the originality of this review is that we highlight the complexity and the specificity of the matrix related to the organ and cancer. Then, we do not only describe a pro-tumor role for ECM but also a protective role, which is less investigated.

## ECM Composition and Its Evolutive Role During Cancer Progression

### Components and Deposition of the Physiological ECM

The ECM and, more globally, the matrisome are dynamic structures composed of thousands of proteins including glycoproteins (such as fibronectin and laminin) and fibrous proteins such as collagens (7). The ECM form structures such as the BM and the interstitial matrix (9). The main role of BM is to act as a physical barrier between the epithelial cells and the stroma of an organ. The BM is more compact than interstitial matrix; it is composed of laminins, heparan sulfate proteoglycans, collagen IV and proteins synthetized and secreted by epithelial cells, endothelial cells and myofibroblasts (9).

The interstitial ECM is mainly composed of collagens I and III, fibronectin, and proteoglycans. The ECM is mainly secreted by fibroblasts, but in different specialized tissues such as cartilage or bones, ECM could be secreted by chondroblasts or osteoblasts, respectively. This physiological ECM is very heterogenous between the organs. For instance, fibroblasts are able to synthetize and secrete collagens I or III, elastic fibers, reticular fibers and proteoglycans, whereas, chondroblasts synthesize and secrete extracellular matrix of cartilage composed of collagen II, elastic fibers and glycosaminoglycans. Osteoblasts synthesize and secrete extracellular matrix of bones principally composed of type I collagen. Specific to blood vessels, different studies showed that pericytes, vascular smooth muscle cells and fibroblasts are able to produce ECM such as collagen IV, fibronectin, and laminin (16). The different origins of these ECM-secreting cells contribute to heterogeneity and complexity of the physiological ECM.

Physiological ECM is constantly remodeled. Indeed, its components are secreted, modified and degraded, in order to adapt and maintain tissue homeostasis. This process is important to maintain physical properties of the different matrix, and also participate in the physiology of the tissue. This remodeling process is deregulated and occurs abundantly during cancer. The ECM in cancer participate in cancer cells epithelial to mesenchymal transition (EMT), BM degradation, migration into the stroma and invasion through the interstitial ECM (17). ECM in cancer is also the interface between tumor cells and normal tissues and could have two opposite roles: protective and pro-tumor.

### Extracellular Matrix Evolution in Cancer

#### ECM Protective Role

The ECM could act as a physical barrier actor in order to inhibit tumor progression. In different cancers, myoepithelial cells or cancer-associated fibroblasts (CAFs) surround the tumor and secrete growth factors, protease inhibitors, angiogenic inhibitors or several tumor suppressors in order to prevent tumor growth, invasion and metastasis. Furthermore, different ECM elements, such as collagen IV or collagen I, could also participate in restraining tumor growth and could first act as a protective barrier by inhibiting cell proliferation. We describe these different elements in detail later in this review.

Very little is known about the protective role of the ECM and how this protective barrier becomes pro-invasive and requires further investigation. Some cancers do not even appear to have any protective effect induced by the ECM or stromal cells. We could hypothesize that when stromal cells are overactivated into stromal cancer cells, they induce an upregulation of ECM component secretion. First, in some cancers, collagen secretion could act as a protective barrier around the tumor cells. Subsequently, cancer cell proliferation and alterations increase over time, the pressure and the stiffness become too high, inducing a stress on tumor cells. To overcome these stresses, tumor cells evolve to pursue proliferation and tumor progression.

#### Tumor-Promoting Role of the ECM

Tumor cells can cause activation of stromal cells into stromal cancer cells that can remodel the ECM to create a pro-tumor environment. We propose to name this matrix promoting tumor progression: Tumor Associated Extracellular Matrix (TAEM). Collagen I is the main component and most studied ECM element, therefore, we focus on this element in this review. Even if the most abundant element of the TAEM is collagen I, ECM is highly complex and heterogenous, and most of the studies of cancers are still mainly performed on only one type of matrix. It is important to study the full matrisome of each cancer and cancer subtype and study the interaction between the different TAEM elements. This would allow better understanding of what role can have each specific molecule. Thus, we could focus on the ones that can have a protective role and could become therapeutic targets.

One general feature during cancer is that type I collagen is overexpressed (18, 19), crosslinked and continuously remodeled, although the process varies between different cancers.

Next, we describe the remodeling of TAEM: (i) its deposition and (ii) its degradation by invadopodia formation (20) through (iii) matrix receptors, leading to loss of ECM homeostasis (19) and change of biomechanical properties of the ECM.

##### ECM deposition

Cancer cells, through activation of normal cells into stromal cells, or by themselves, can remodel physiological ECM into TAEM. Fibroblasts are the most abundant cells of the tumor stroma and are involved in several biological processes. Some fibroblasts can be recruited, activated and transformed into CAFs by different secreted factors from tumor cells in the microenvironment such as TGF-β, PDGF or FGF (21, 22). CAFs can result from the activation of fibroblasts near the tumor, mesenchymal stem cells, but also from cells that have undergone EMT (21–26). Once activated, owing to their different origins, CAFs possess a variety of tumor promoting functions, adding another step in the complexity and heterogeneity of the stroma.

One of their functions is to secrete various ECM elements including collagen I, fibronectin and hyaluronan, growth factors (HFG, PDGF, and CTGF), chemokines, cytokines, interleukines (IL-6 and IL-8) and proteases in order to promote tumor cell proliferation, angiogenesis and invasion (3, 27). Moreover, during remodeling in cancer, ECM undergoes drastic structural changes due to chemical and physical restructuration, leading to TAEM. Many studies have shown an increased ECM deposition, inducing a stiffer stroma; in addition, morphological changes that occur are characterized by more aligned collagens at the tumor front (28). Tissue stiffness can be increased by enzymes such as lysyl oxidases (LOX), which can crosslink collagen. These enzymes can be secreted either by stromal or tumor cells, inducing increased crosslinks and, thus, an accumulation of collagen I, fibrosis and promoting metastasis (29–31).

Different studies showed mechano-regulatory mechanisms wherein ECM rigidity perturbs epithelial morphogenesis and tissue polarity (28, 32–34). For example, Weaver et al. have shown that this mechanism will enhance ERK activation and increase cancer cell malignant phenotype (28). CAFs can also mechanically remodel the ECM, through compaction and CAF contractility, in order to create paths to increase cancer cell migration and invasion (35).

##### Invadosome formation leads to ECM degradation

The other way to remodel the ECM into TAEM is by degradation. This ECM degradation can be achieved by cancer cells and all cells present in the tumor microenvironment, all of them can form invasive and degradative structures called invadosomes. Invadosomes are membrane protrusions that can be found on normal cells (named podosomes) as well as in tumor cells (where they are named invadopodia). Contrary to other actin-based structures such as filopodia, focal adhesions or lamellipodia, invadosomes not only possess adhesive, mechanosensitive capacities but also proteolytic activity by recruiting, secreting and activating matrix metalloproteinases (MMPs), allowing them to degrade the ECM. They also present their own translational machinery to maintain their structure and function (36).

Invadosomes are plastic structures, with the ability to adapt to the available ECM receptors as well as to the microenvironment. Invadosomes are complex and highly dynamic structures composed of a F-actin core surrounded by a ring of scaffold and adaptor proteins in 2D. Actin-regulating proteins, kinases and small GTPases regulate actin machinery within the invadosomes (37, 38). Key molecular players for functional invadosomes have already been identified, including the adaptor protein Tks5, Cdc42 (36, 39), the actin regulators cortactin and N-WASP, as well as the transmembrane protein MT1-MMP (37).

Even though invadosomes share a common molecular signature, they exist in different organizations, depending on the cell type and on the microenvironment. Cells can form invadosomes as dots (such as MDA-MB-231 cells), as rosettes (such as NIH3T3-Src cells) or as aggregates (such as macrophages and osteoclasts). All of them can reorganize their actin cytoskeleton to form another class of invadosomes, called linear invadosomes, when seeded on type I collagen (40, 41).

This last linear organization is induced by physiological fibrillar collagen I and form specifically along fibrils. Even if cells can form invadosomes to degrade the BM, this suggests that when cancer cells are in direct contact with collagen I after BM degradation, TAEM promotes invadosome formation. The collagen receptor discoidin domain receptor 1 (DDR1) is responsible for linear invadosome formation and their degradation function. Indeed, DDR1 activates the RhoGTPase Cdc42 and its guanine exchange factor Tuba, inducing their localization in linear invadosomes (42).

Moreover, other collagen receptors such as integrins or CD44, that can also be found on stromal cells as well as cancer cells, have also been shown to be involved in invadosome formation (43–45). Most cells possess the ability to form invadosomes that are dependent on various stimuli like growth factors (VEGF, TGF-β…), genome alteration or microenvironment (40, 45, 46), allowing TAEM degradation.

We can hypothesize that cell cooperation between cancer cells and stromal cells could promote invadosome formation: indeed, stromal cancer cell activation by tumor cells induce ECM deposition and secretion. This will, in turn, promote invadosome formation by the binding of ECM elements (such as collagen I) to cancer cell receptors. Different studies have shown that increased ECM rigidity promotes invadosome formation and activation. Some studies already demonstrated a cooperation between tumor cells and CAFs or macrophages in order to secrete ECM-degrading enzymes (47–50), but no study clearly demonstrated cell cooperation to directly promote invadosome formation. However, we could imagine that stromal cells around the tumor, such as fibroblasts or endothelial cells, could secrete many soluble factors such as TGF-β or TNF-α in order to promote invadosome formation by cancer cells. This would lead to an invasive loop, inducing TAEM degradation, at the same time as tumor cell proliferation and angiogenesis.

To sum up, both stromal and cancer cells are able to create TAEM by secreting ECM and degrading it to promote tumor growth, invasion and metastasis. To create TAEM, a crosstalk between stromal and cancer cells is needed. This TAEM will, in turn, serves for communication between stromal and cancer cells. In order to mediate the interaction with the TAEM, stromal and tumor cells will bind with different matrix elements via the presence of receptor panels on their surface, each cell expressing different receptors modulated during tumor progression, contributing to tumor heterogeneity.

##### ECM receptor expressions in cancer cells and in CAFs

Even though many receptors are able to bind the ECM, three are mainly described in tumor progression (CD44, integrins and DDRs). Due to prominent interest in cancer cells, rather than in stromal cells and ECM, a large number of well-described reviews focus on these matrix receptors in cancer cells (14, 51–53). Indeed, we describe, the role of CD44, integrins and DDRs - notably in invadosomes and metastasis formation - in cancer cells and CAFs.

CD44 is a transmembrane glycoprotein receptor which is an adhesion molecule that is upregulated following tissue injury, and is implicated in many chronic inflammatory diseases such as atherosclerosis or autoimmune diseases. It can interact with its extracellular domain with different ligands like HA, osteopontin, fibronectin, collagen, MMPs and different growth factors such as HGF, bFGF and VEGF. This receptor is overexpressed in CAFs (54) and in a large number of cancer cells [pancreatic cancer, breast cancer, prostate cancer, head and neck squamous cell carcinoma (HNSCC), and gastrointestinal cancer] where it is involved in several steps of tumor progression such as tumor invasion, EMT, metastasis formation and resistance to chemotherapy (52). High expression of CD44 in cancer cells is also associated with cancer stem cell (CSC) properties and is used as a CSC marker. CD44^+^ cancer cells show an increase in EMT and in invasion, correlated with poor prognosis (55–58).

Integrins are transmembrane heterodimers which consist of α-subunit associated with a β-subunit in a non-covalent manner. Integrins are able to bind different elements of ECM such as vitronectin, fibronectin, laminin or collagens. Only four integrins are able to bind collagen I: α1β1, α2β1, α10β1, and α11β1 (59). Integrins are overexpressed in a large number of cancers in both stromal and tumor cells where they can promote survival, proliferation, motility, invasion, and ECM modulation (53). Moreover, various studies have shown that integrin α11 is expressed in CAFs in a large number of cancers, like non-small cell lung cancer (NSCLC) or HNSCC. In these cancers, α11β1 expression is involved in migration, tumorigenicity and invasion of tumor cells (60–63). Furthermore, in NSCLC, α11β1 expressed in CAF induces collagen reorganization and tissue stiffness, promoting tumor growth and metastatic potential of tumor cells (63). Thus, α11β1 seems to be an important receptor for collagen remodeling and CAF migration in the tumor microenvironment.

DDRs are members of the tyrosine kinase receptor family and are composed by two members, DDR1 and DDR2 (64). These transmembrane receptors are activated by collagens in their native triple helix form (65–67). Moreover, DDRs are involved in several physiological functions such as embryogenesis and wound healing and are overexpressed in a large number of cancer subtypes, where they are associated with cell proliferation, invasion, migration and drug resistance (51).

DDR1 and DDR2 play an important role in the tumor microenvironment which is involved in the dissemination of tumor cells. These receptors could be expressed both by cancer cells and CAFs in order to promote tumorigenesis. For instance, Jin et al. have shown that CAFs promote the secretion of cytokine IL-6 which activates the JAK/STAT3 pathway in gastric carcinoma cells, inducing DDR1 upregulation, promoting peritoneal tumorigenesis (68). Thus, inhibition of DDR1 is an interesting strategy for the treatment of peritoneal metastasis of gastric cancer.

To sum up this part, tumor cells are able to activate fibroblasts into CAFs by factor secretion and CAFs are in turn able to secrete TAEM in order to promote tumor cell invasion, proliferation, migration and metastasis. To our knowledge, little is known about the effect of these collagen receptor expressions in other stromal cells such as immune cells or adipocytes and on the crosstalk with tumor cells.

However, these collagen receptors are known to be involved in invadosome formation, allowing tumor cells to remodel and degrade the ECM in order to migrate, invade and form metastasis. Those receptors are also able to interact together (DDRs/integrins and integrins/CD44) (64, 69, 70). It will be important to study if these three receptors cooperate together in tumorigenesis and if there is any compensation in their functions.

Although the remodeling of the matrix is an important step, this classical model of ECM remodeling does not apply to all cancers. Indeed, this model of an increased ECM deposition, stiffness and increased activated stromal cells, neither considers the complexity of the organ, the heterogeneity of the tumor nor the specificity of its own ECM. Moreover, most studies focus on the pro-tumor role of ECM whereas initially, in certain cancers, it could play a protective role, making it possible to restrict tumor progression. Then, the dynamic of the microenvironment causes the protective side of the matrix to become pro-tumor. This will in turn induce ECM rigidity, remodeling and degradation which will then promote tumor cell invasion (9, 12).

To illustrate this point, we next describe the composition and evolution of the ECM in five cancers: breast cancer, liver cancer, pancreas cancer, colon cancer and melanoma as well as the dual role of their ECM in cancer progression.

## Breast Cancer

### ECM Composition and Function

Healthy breast epithelium forms a ductal network surrounded by adipose tissue. This network connects mammary lobes to nipples. The normal breast tissue is made of two compartments: the epithelium and the stroma. The epithelium of the ducts and of the lobule of the mammary gland is made of luminal cells, which express hormone receptors, and myoepithelial cells. Both cell types are surrounded by a BM. The mammary gland goes through several cycles of changes such as differentiation, development and apoptosis during physiological adult life, including during puberty and pregnancy (71–73). These cycles are highly regulated, but the disruption of the tissue homeostasis, tissue organization and cell function can lead to cancer.

The most common breast cancer is ductal carcinoma. It is thought to arise after cellular abnormalities, inducing abnormal proliferation in the terminal duct lobular units. Then, a multistep transformation of epithelial cells and accumulation of abnormalities induce hyperplasia, premalignancy, *in situ* carcinoma, and finally, invasive carcinoma (71, 72). Breast cancers are highly heterogenous and are divided into six subtypes, depending on their histology, epidemiology and molecular signatures: luminal A, luminal B, Her2-positive, claudin-low triple negative (also called basal-like), and normal-like (73). Their diversity induces more or less invasive forms with different clinical outcomes.

**FIGURE 1:**
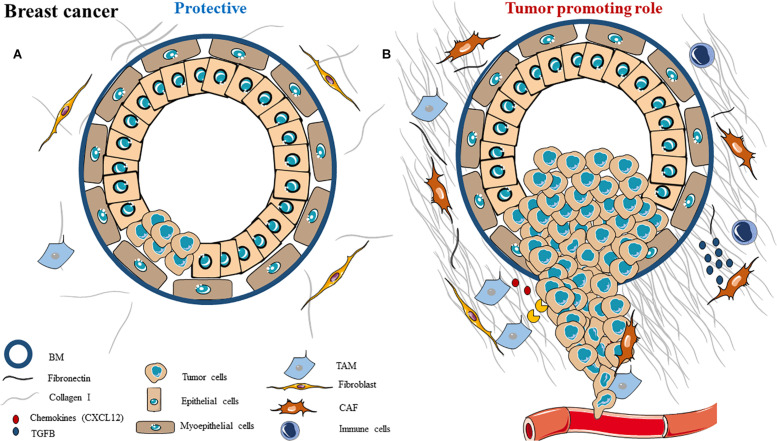
Schematic representation of ECM composition and ECM dual role as a **(A)** protective barrier or as a **(B)** tumor promoting role in breast cancer.

The tumor microenvironment of breast cancer is far from homogenous and can evolve during tumor progression in the same tumor (Figure 1). From primary tumor growth to extravasation and metastasis formation, the ECM is constantly changing. For example, even when a ductal carcinoma *in situ* (DCIS) becomes an invasive carcinoma, the microenvironment is different, due to a differential gene expression of all the cell types between these two cancer steps (74). The ECM is highly dynamic and is now known to be a major player in tumor progression (75).

The ECM in breast shows similarities to tissues undergoing wound healing (76, 77) or breast tissue going back to homeostasis through remodeling after pregnancy, with overexpression of fibrillar collagens, fibronectin and ECM remodeling enzymes (78). This change in ECM has also been associated with increased risk of breast cancer after pregnancy (78, 79).

During breast cancer, one main change in the ECM is the collagen abundance (Figure 1). Collagen I, III and V are accumulated while collagen IV is decreased, due to degradation of the BM (75). Collagen crosslinking is increased too, inducing a change in collagen organization (shaping it more aligned), and an increased ECM stiffness. Both characteristics are associated with tumor progression. The crosslinking is facilitated by LOX enzymes, which are also overexpressed in breast cancer (29, 80, 81).

Collagen fibril formation is induced by fibronectin (82), changing collagens into scaffold for tumor cells to migrate and invade (48). Fibronectin is also overexpressed during breast cancer (by CAFs and cancer cells) and is associated with poor prognosis, notably because it promotes metastasis (83, 84). Hyaluronan as well as versican also accumulate in the breast cancer ECM and are associated with poor prognosis (85, 86). Indeed, hyaluronan helps creating a pro-tumor microenvironment (87), while versican promotes breast cancer cell self-renewal and migration (88, 89). Several matricellular proteins, such as osteopontin, tenascin C or periostin, are also overexpressed during breast cancer and are associated with increased migration, invasion, and a poor outcome (90).

Extracellular matrix modifications do not only come from matrix components, but also from remodeling enzymes: from proteases such as MMPs (MMP-2, -3, -9, and -14) to crosslinking enzymes such as LOX. These enzymes are often overexpressed in breast cancer, and promote cancer development and metastasis (76). However, these two families of enzymes can be differentially expressed depending on cancer subtypes. For example, LOX, LOXL2, LOXL3, and LOXL4 are overexpressed in more invasive cancers, such as triple negative breast cancers, inducing cancer cell invasion and metastasis (91, 92). Similarly, MMP-9 overexpression is higher in high-grade and more invasive breast cancers (such as triple negative and Her2-positive), where it is associated with metastasis and relapse (93).

### ECM Evolution During Cancer

#### Protective Role

Several components of the ECM can first have protective roles in order to inhibit tumor progression (Figure 1A). For example, myoepithelial cells can be considered as the main natural tumor suppressor in breast cancer, and their disruption seems to be a key step in tumor progression. Indeed, the myoepithelial cells are located between the stroma and the luminal cells (from which cancer arises), creating a separating sheet between the epithelium and the stroma. They have an important role during lactation as well as protective roles during tumorigenesis, as they form a physical barrier around luminal cells (94–96). Myoepithelial cells can act on tumor cells and on fibroblasts to reduce MMP-2, MMP-9, and MT1-MMP gene expression, decreasing cancer cells invasive capacities (97). They also express some proteinase inhibitors, such as the MMP inhibitor TIMP-1, and angiogenic inhibitors such as thrombospondin-1 and bFGF receptors (98), allowing them to inhibit angiogenesis (99). They can secrete several tumor suppressors such as maspin, cytokeratins, relaxin and activin in order to prevent tumor growth, invasion and metastasis (96). The myoepithelial cells also participate in accumulating ECM and basement membrane instead of degrading it. To do so, these cells express high levels of collagen, fibronectin and laminin (100, 101). All of these show that these specific cells can have several positive roles in preventing tumorigenesis.

Moreover, some studies also suggest a protective role for CAFs in breast cancer. CAFs can secrete factors such as caveolin-1 and podoplanin, which are associated with decreased metastasis (102). CAFs can also inhibit PI3K and TGF-β signaling through secretion of SLIT2 and asporin, respectively, inducing a decrease in EMT, invasion and metastasis (103).

Proteoglycans are proteins that are heavily glycosylated and can bind ECM components like collagens. Decorin, a member of the proteoglycan family, is also known to have anti-tumor roles (104). Indeed, reduced expression of decorin is associated with poor prognosis and may promote tumorigenesis and invasion (105), while its overexpression is associated with better prognosis and leads to tumor growth and metastasis inhibitions (through ERbB2 inhibition) (106–108).

This suggests that several cell types and ECM elements may have protective roles in breast cancer, but some of them may not be elucidated yet, and it needs further investigation. However, there is not enough information to understand at what stages stromal cells are activated and when the protective role becomes pro-tumor. It would be important to understand this time frame in order to block this transition to inhibit tumor progression.

#### Tumor Promoting Role

Many ECM components (cellular as well as matrix) play a role in favoring breast cancer progression (Figure 1B). For example, CAFs are the most abundant cell types in breast cancer stroma, they can derive from resident fibroblast or myoepithelial cell activation (103). CAFs can secrete ECM components (such as type I collagen or fibrin) and several soluble factors, such as growth factors (EGF, HGF, TGF-β), metalloproteinases (MMP-1, -2, -9) or chemokines (CXCL12), to promote tumor growth and metastasis (74, 95). Macrophages (also known as tumor-associated macrophages, TAMs) are also involved: they can secrete VEGF, cytokines or TGF-β to promote cancer cell survival, angiogenesis and invasion (109, 110). Finally, tumor-infiltrating lymphocytes help tumorigenesis by blocking anti-tumor response and suppressing immune cells (111).

Several studies showed that myoepithelial cells from normal or cancer tissues strongly differ in their gene expression. The cells isolated from normal tissue express high levels of interstitial ECM, such as laminin, tenascin or tropomyosin, while the cells isolated from DCIS overexpress proteases (such as MMP-2), protease inhibitors (such as TIMP3 or thrombospondin-2), chemokines, cytokines and collagens (74). They are also deficient in production of laminin, showing that they tend to degrade the normal ECM instead of depositing it as in physiological conditions (72). Another study by Hu et al. showed that myoepithelial cell differentiation must be maintained in order to avoid invasive phenotype of breast cancer. Loss of myoepithelial cells, through inhibition of TGF-β, Hedgehog, p63 or cell adhesion signaling by tumor cells induces the transition from DCIS into invasive carcinomas, suggesting that loss of myoepithelial cells is a prerequisite for tumor invasion (112).

Moreover, increasing invasion and metastasis can also be promoted through a crosstalk between different cell types. For example, Condeelis et al. have shown, using intravital imaging, that tumor microenvironment plays a key role in invasion and metastasis by creating an essential paracrine loop between tumor cells and macrophages with direct interaction of the two cell types. This induces a specific microenvironment, dependent on macrophages and EGF and CFS-1 signaling, which is essential for intravasation of cancer cells (48).

Extracellular matrix binding receptors are also involved in this tumor-promoting role. Indeed, CD44, integrins and DDRs are overexpressed in breast cancer and promote tumor progression (51–53, 64). For example, CD44 standard isoform (CD44s) is positively correlated with CSC gene signature in breast cancer, notably through PDGFRβ/Stat3 activation (113). CD44 can also activate several signaling pathways such as MAPK, PI3K/Akt to induce migration, survival and invasion (114).

Integrins are also key players in breast cancer, notably in the metastatic cascade. Indeed, they promote migration, MMP expression, secretion and location at invadosome in order to facilitate invasion (115). They also directly control invadosome formation and can be found localized in these structures (116). Moreover, one study demonstrated that collagen binding integrin α11 expressed by CAFs activates PDGFRβ/JNK signaling in breast cancer cells to promote tumor cell invasion (117).

Concerning DDRs, Corsa et al. demonstrated that in CAF, DDR2 is critical for ECM production and the organization of collagen fiber (118). They also showed, in these cells, that DDR2 is involved in breast cancer cells metastasis in the lungs, by affecting collective cell migration. Furthermore, this team demonstrated that DDR2, when expressed by stromal cells, promotes the metastatic spread of breast cancer cells. DDR1 has also been shown to be involved in many steps of breast cancer, including invasion (through its interaction with collagen I and invadosome formation), proliferation, migration (both through its association with the insulin-like growth factor-I receptor) and resistance to treatment (through its interaction with collagen IV and NFκB activation) (42, 51).

Concerning the matrix components (secreted by cancer cells as well as stromal cells), fibronectin overexpression can modulate cancer cell signaling in order to promote tumorigenesis, for example, by inducing EMT via ERK (119) or STAT3 (120) activation. Laminins are also involved: laminin-5 can promote survival through NFκB activation in activated B cells (121) and invasion and migration through integrin interaction (122), and laminin-511 promotes metastasis (123). Versican can also increase tumorigenesis by inducing cancer-cell self-renewal through EGRF signaling (89) and by inducing cell survival, tumor growth and metastasis (124, 125).

Collagen is also described as a key player in tumor development. The increased ECM stiffness during cancer induces a change in biochemical signaling and in cell behavior, promoting tumor progression in several ways. For example, increased stiffness in mammary epithelial cells induces MAPK activation and proliferation (126). This mechano-regulatory mechanism could also induce aggressive phenotype in tumors (28). Increased stiffness of ECM also promotes transcriptional coactivator with a PDZ-binding motif (TAZ) activity (leading to an increase of CSC properties) (127) as well as PI3K activity (leading to invasion) (80). Studies have shown that, to increase invasion, matrix density can also promote invadosome formation and ECM degradation (128). Invadosome formation can also be induced by the ECM itself, via collagen: type I collagen is an inducer or linear invadosome formation and matrix degradation (41, 42). Indeed, breast cancer cells seeded on type I collagen tend to have an increased matrix degradation capacity than on gelatin.

Extracellular matrix degradation is mediated by proteases. In cancer, MMPs are key players in ECM remodeling and degradation. Some of them, such as MMP-2, MMP-9 and MMP-14 are overexpressed in breast cancer, inducing collagen degradation and promoting metastasis (129, 130). Heparanase, another ECM remodeling enzyme, has been shown to be involved in breast cancer progression. Its overexpression induces mammary tumor growth, survival and cell spreading (131–134). Similarly, the inhibition of cathepsins, which are lysosomal proteases, was shown to inhibit breast cancer metastasis (135, 136).

To sum up, in breast cancer, many ECM players are involved in tumor progression, creating stroma that are either pro-invasive or protective. However, studies we reviewed did not specify differences between breast cancer subtypes, because they are mostly performed with the same types of samples (MDA-MB-231 or MCF-7 cells *in vitro*, and comparing normal breast and DCIS *in vivo*). There is a real need to find new matrix to work on (not only collagen matrix), and to work in 3D using organoids, adapted to each cancer type and subtype, to be more representative of what is happening for real *in vivo*.

## Liver Cancer

### ECM Composition and Function

Liver is structured in highly organized units of hexagonal shape called lobules, whose size is about 1 mm. The prominent cell type (50–60% in cell number) is hepatocytes (parenchymal cells), which carry out the main functions such as detoxification, synthesis of plasma proteins, lipids, glycogen, and activation of inflammatory or immune responses. However, about 40% of the liver cells are non-parenchymal (NP), including sinusoidal endothelial cells (LSEC, serve as a filtration barrier), Kupffer cells (KC, function as *in situ* macrophages), hepatic stellate cells (HSC, fat-storing cells; play a major role in the progression of fibrosis) and a small fraction of biliary epithelial cells (cholangiocytes) and liver-associated lymphocytes and leukocytes.

Besides this diversity in cell types, the population of hepatocytes is itself heterogeneous: hepatocytes are functionally different depending on their location within the lobule, dictated by the unique vasculature of the liver. Perivenous (or centrolobular) hepatocytes are exposed to lower oxygen tension as well as nutrient and hormone levels. In other words, the oxygen gradient through the lobule translates into a gradient of metabolic functions, which leads to the so-called zonation of the liver (137). In adults, normal liver ECM is mainly composed by collagen (60%), non-collagenous proteins and proteoglycans. Collagen I (COL1A1 and COL1A2) is predominant, but other collagens such as COL2A1, COL21A1, COL23A1, COL5A3, and COL26A1 are present. Collagen fibers were found in the portal tracts, whereas the normal parenchyma contains only few collagen fibers (138). An originality of liver microvasculature is the presence of a very fine and partial basal membrane associated with fenestrated endothelial cells to facilitate exchange between blood and hepatocytes.

Liver cancers are the fourth most lethal cancers worldwide (139). Hepatocellular Carcinoma (HCC) is the most common form of primary liver cancer. Intra- and extra-hepatic metastases are usual complication in HCC. Due to frequent late diagnosis, the prognosis for HCC is poor. In most cases, HCC develops upon chronic liver disease caused by various factors such as viral hepatitis B/C, alcohol or metabolic syndrome (Non-Alcoholic SteatoHepatitis). Persistent hepatic injury and associated regeneration could produce a stressful environment leading to inflammation and hypoxia, which are features of HCC microenvironment.

In most cases (70%), HCC occurs on a cirrhotic liver. Cirrhosis is characterized by formation of regenerative nodules of liver parenchyma that are separated by fibrotic septa. Activation of hepatic stellate cells (HSCs) into myofibroblasts, mostly characterized by Smooth Muscle Actin (SMA) expression are the principal source of secrete matrix playing an important role in the initiation of liver fibrosis, cirrhosis development and cancer emergence. In normal liver, HSCs are quiescent cells found in the perisinusoidal space of Disse. Chronic liver injuries promote a complete cell transdifferentiation into proliferative myofibroblasts. In this context, the microenvironment is very specific, associated with type I and type II collagens and elastin accumulation corresponding to the pathological evolution of liver fibrosis.

Nevertheless, in some cases, HCC is observed in non-pathological liver. Consequently, the matrix microenvironment varies a lot between the different HCCs in terms of etiology and the presence of cirrhosis or not. Here, we describe the role of ECM on HCC progression and invasion.

**FIGURE 2:**
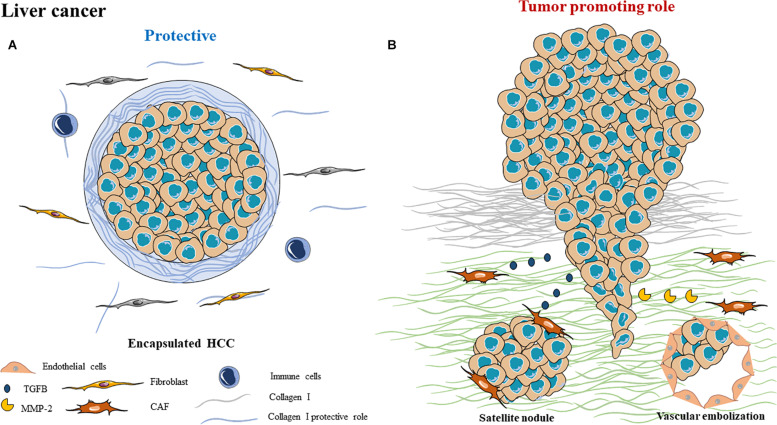
Schematic representation of ECM composition and ECM dual role as a **(A)** protective barrier or as a **(B)** tumor promoting role in liver cancer.

Some ECM elements are deregulated during cirrhosis and HCC. Those ECM elements can be secreted by different cell types such as tumor cells and myofibroblasts or CAFs. Several matrix elements such as type I and type III collagens are upregulated during fibrosis and cirrhosis. In HCC, other matrix elements such as type IV collagen, tenascin, osteopontin and laminin are upregulated (Figure 2). In normal liver, heparan sulfate is the main glycosaminoglycan component, whereas chondroitin sulfate is prevalent in HCC.

Various proteoglycans (PGs) are involved in HCC progression, at cell surface (such as syndecan-1 or Glypican 3), in the pericellular space (such as agrin or collagen XVIII/endostatin) and in the extracellular space (for instance versican, decorin). Most of these PGs are overexpressed in HCC and can serve as biomarkers (140).

### ECM Evolution During Cancer

#### Protective Role

In a significant proportion (40 to 60%), HCC can be surrounded by a fibrous capsule, whose thickness varies from 0.13 to 3 mm (141), presenting a trabecular pattern (Figure 2A). This encapsulation is present in small (≤5 cm), as well as in large HCC (>5 cm) (142). There is no link between the presence of a capsule and the presence of cirrhosis. It is important to note that several studies have shown that this fibrous capsule is associated with a better prognosis than non-encapsulated tumors, suggesting a protective effect of this capsule (142). On the contrary, the presence of an invaded capsule corresponds to a bad survival prognosis, a recurrence and a non-transplantation criteria (143).

This capsule is composed of several matrix elements, including type I and III collagens (144) and the presence of an inflammatory infiltrate is not systematic. To date, there are only few studies on the molecular mechanisms that control the formation of this capsule and the cellular origin of the elements that compose it. A study by Ishizaki et al. demonstrated the presence of positive α-SMA cells, which is a marker of CAF, associated with the presence of procollagen I and III in the capsule (145). The origins of CAFs can be multiple, contributing to the heterogeneity of the tumor. They could participate in the secretion of this fibrous capsule in collaboration with myofibroblasts.

Most analyses of this capsule are based on the immunohistochemistry technique. New global studies could allow further the knowledge of the composition of this structure and determine molecular mechanisms and ECM elements associated with the protective effect of the capsule.

#### Tumor Promoting Role

In presence or absence of a capsule, HCC is a highly invasive tumor (Figure 2B). HCC invasion criteria correspond to satellite nodules, vascular embolization and are hallmarks of HCC progression. Intra-liver metastasis formation contributes to the very high HCC mortality rate as they cause liver failure. Presence of these invasive features is a non-transplantation criterion, which is the only way to treat advanced HCC.

Hepatocellular Carcinoma tumors often occur in cirrhosis context where the number of activated fibroblasts is very high. Several studies have shown the importance of crosstalk between cancer cells and fibroblasts in HCC. Cytokines secreted by cancer cells, such as transforming growth factor-β (TGF-β), stimulate myofibroblasts, leading to their activation. Growth factors and inflammatory cytokines such as PDGF, TGF-β, TNF-α, IL-6, and IL-1β, expressed by cancer cells during HCC, activate and transform quiescent fibroblasts into myofibroblasts and then into CAFs (146). Several studies demonstrate the role of CAFs during HCC progression. A positive correlation exists between the frequency of CAFs around HCC nodules and the tumor size. Moreover, these cells secrete the hepatocyte growth factor (HGF), fibroblast growth factor (FGF), TGF-β, CCL-2, -5, -7 and CXCL16, promoting tumor cell proliferation and invasion, respectively (147).

Increased expression of MMPs was detected at the nodule periphery; metalloproteinases such as MMP-9, MMP-2 and MT1-MMP are probably involved in HCC invasion. Indeed, TGF-β is overexpressed and overactivated during HCC, inducing an increase in ECM deposition (such as type I collagen) and EMT (148). LOXL2 is also a very important element in HCC, its expression is controlled by hypoxia and TGF-β. LOXL2 modulates matrix rigidity, increasing collagen crosslinking and promoting invasion (149). Matrix accumulation and crosslinking increase stiffness, inducing HCC cell proliferation and invasion (150). Physical parameters seem to be crucial to promote HCC progression. Indeed, if the fibrous capsule plays first a protective role, its rigidity could then promote an invasive switch. To illustrate this point, an invaded capsule corresponds to a very aggressive feature associated with a very poor prognosis.

Hepatocellular Carcinoma invasion can be increased by different ways. Indeed, EMT, MMPs secretion and matrix stiffness are elements that control invadopodia formation. Several studies have demonstrated the ability of HCC cells to form invadopodia and to degrade ECM. Keratin 19, MMP-2, TIMP2, Mena, Agrin, Src, and TGF-β are notably described to participate in invadopodia formation in HCC cells (151–154). For example, TGF-β stimulates type I collagen, DDR1 and LOXL2 expression, modulating ECM organization and inducing invadopodia formation (155).

Accumulation and overexpression of various ECM elements also promote cell proliferation, provide survival signals and induce tumor invasion. In parallel, associated receptors must be present and are involved in signaling pathways. In fact, in HCC, a large number of ECM receptors are overexpressed such as integrins, CD44, DDRs. For example, β1 integrin induces a pro-survival signal through MAPK pathway in HCC cells (156). CD44 plays an important role in tumor cell initiation, proliferation, invasion and CSC properties (157). CD44 is required for Mdm2 nuclear translocation and AKT activation leading to tumor progression (157).

Discoidin domain receptor 1 and DDR2 are also overexpressed in HCC. Both participate in tumor cell proliferation, EMT and invasion processes through ERK signalization, SNAIL1 stabilization and MMPs activation, respectively (155, 158, 159).

To conclude, a large number of studies demonstrate a real impact of the ECM on the development and evolution of HCC. However, many questions remain. Moreover, this notion of protection or, on the contrary, pro-invasive role of the ECM is not yet considered in the clinic, neither in the diagnosis nor in the management of the patients. This aspect is obscured not only by the lack of knowledge but also by the lack of adapted therapeutic solutions. At the research level, *in vivo* and *in vitro* models do not reflect the complexity and dynamics of the interface between the tumor and the ECM.

## Pancreatic Cancer

### ECM Composition and Function

**FIGURE 3:**
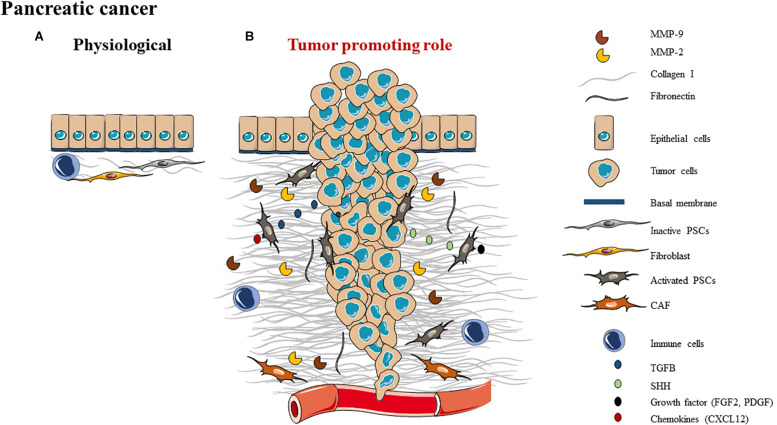
Schematic representation of ECM composition **(A)** and ECM role as a **(B)** tumor promoting role in pancreas.

In physiological conditions of pancreas, BMs predominate, occurring around each acinar cell of the exocrine pancreas, surrounding blood vessels and encasing each pancreatic islet (160, 161). The interstitial matrix confers tensile strength and elasticity to tissues, mainly due to the presence of fibrillar collagens. The interstitial matrix is limited in the pancreas and appears as a thin layer immediately subjacent and external to the peri-islet BM and surrounding large ducts and blood vessels. One specificity of the pancreas ECM it is that there is no hyaluronan, but it is composed of hyaladherins such as versican, inter-alpha-inhibitor (IαI), and tumor necrosis factor-stimulated gene-6 (TSG-6) (162). The human peri-islet BM is mainly composed of collagen type IV, agrin, perlecan, nidogen-1 and -2 and laminin isoforms (160, 161). In normal pancreatic tissue, resident fibroblasts, pancreatic stellate cells (PSCs), immune cells, and vascular cells play a critical role in tissue repair and wound healing (163) (Figure 3A). In physiological conditions, quiescent PSCs reside at the basolateral aspect of pancreatic acinar cells and could synthesize ECM proteins and ECM degrading enzymes (164). Following pancreatic injury or tissue damages, injured acinar cells produce and secrete inflammatory cytokines and pro-angiogenic growth factors that increase recruitment and activation of immune cells, promoting angiogenesis. This also leads to increased PSC-mediated deposition of ECM to restore normal pancreatic function. PSCs regulate ECM by maintaining the balance between ECM synthesis and degradation (165, 166).

Pancreatic ductal adenocarcinoma (PDAC) is the most common type of pancreatic cancer. In PDAC, disruption of BM integrity leads to a decrease of collagen IV, altered epithelial cells become cancer cells and activate PSCs to create a permissive microenvironment for cancer progression (167). Once PSCs are activated, the equilibrium shifts, that causes ECM proteins such as collagen I to accumulate (164, 168). This abundant amounts of ECM corresponds to a desmoplastic reaction which exerts mechanical and biochemical effects of PDAC cells by promoting tumor progression (168). The PDAC fibrotic stroma is composed of connective tissues which are rich in collagens I (mainly) and III, fibronectin, CAFs [most of them are pancreatic stellate cells (168)], vascular and immune cells as well as cytokines and growth factors (169–173) (Figure 3B).

### ECM Evolution During Cancer

#### Protective Role

The vast majority of patients with PDAC present metastatic disease whereas, normally, deposition of huge amounts of collagen around PDAC cells might inhibit invasion and metastasis. Indeed, PDAC cells have mechanisms that help them overcome this fibrotic barrier and ECM here provides a protective effect in PDAC. Therefore, to our knowledge, there is no physical barrier mediated by the ECM or stromal cells that could constrain tumor progression. However, some matrix elements could be involved in a protective role and are described as better prognosis in PDAC. Indeed, overexpression of some components of the ECM such as collagen XV could act as a tumor suppressor in the BM zone by reducing migratory ability of PDAC cells (174). Proteoglycans can be expressed by tumor cells as well as stellate cells and could play anti-tumor role. For example, biglycan expression is inversely correlated to poor prognosis (175). For instance, lumican expression is associated with an increased survival in patients. It is expressed in both the tumor and the stromal compartments and could directly interact with tumor cells, turning PDAC cells into quiescent cells in G0/G1 arrest (176).

#### Tumor Promoting Role

The ECM is essential in PDAC development, from the initiation to tumor progression (Figure 3). The fibrotic ECM tumor stroma is mainly composed by CAFs and most of them are pancreatic stellate cells (168). PDAC cells secrete Sonic Hedgehog signaling molecule and TGF-β to attract and activate PSCs. Activated PSCs produce pro-inflammatory growth factors and chemokines which could act as a feedback loop to maintain their activity and then promote the synthesis of ECM proteins such as collagen (177–179). Subsequently, activated PSCs promote tumor growth and local invasion of PDAC cells (180).

Pancreatic ductal adenocarcinoma cell properties could also be altered by tissue stiffness of the ECM, which reduces tissue polarity, inhibits adherent junctions, promotes tumor cell proliferation and EMT, by altering expression of vimentin and E-cadherin in PDAC cells (181). Inhibition of PDAC cell contractility decreases MMP activity, suggesting that PDAC cells also influence the ECM properties (182). Crosslinking of collagen I in PDAC could be mediated by LOX and tissue transglutaminase 2 (TG2) (31, 183, 184). TG2 is weakly expressed in normal pancreatic tissue, but its expression and secretion in ECM are increased in PDAC cells (56). Crosslinked collagen activates Yes-associated protein (YAP) and TAZ and promotes proliferation and EMT of PDAC cells (56). ECM degradation is mediated by proteases. For instance, in PDAC, MMPs are key players in ECM remodeling and degradation, as well as in proliferation of Panc-1 cells (185). One study showed that ROCK1 and ROCK2 promote expression of MMP-10 and -13, enhancing collagen degradation and thus local invasion (186).

Some other matrix components play a crucial role in promoting tumor progression. In patients with PDAC, a level of laminin inferior to 25% in BM (due to BM disruption) or an increase of circulating collagen IV are associated with bad prognosis (187, 188). In PDAC, fibronectin shares similarities with collagen: it can also bind to integrins (such as α5β1) leading to FAK activation (189). Fibronectin acts as a major pro-tumor actor in PDAC, promoting resistance to radiotherapy, proliferation and production of reactive oxygen species (190, 191). Fibronectin also plays an important role in amplifying ECM synthesis by PSCs. By binding to the latent TGF-β binding protein, fibronectin allows the release of active TGF-β, which in turn activates PSCs (192). Similar to fibronectin, vitronectin is a major glycoprotein that binds to both integrins (α5β3) and collagens (193). In physiological conditions, vitronectin is involved in wound healing and homeostasis whereas in PDAC, vitronectin is overexpressed and binds to collagen I, promoting cancer cell migration. It also stimulates secretion of interleukin 8 and promotes proliferation of PDAC cells (194, 195). Proteoglycans such as Glypican-1 is overexpressed in PDAC tumor cells and involved in tumorigenicity (196). Another proteoglycan, SPOCK-1, is able to remodel the ECM, and allows tumor cells to become more invasive (197). HA, which can bind to proteoglycans, is important to promote cell survival, proliferation, and invasion through its binding to CD44 and to the receptor for HA-mediated motility (RHAMM). HA is required, with the help of collagen, to induce an increase in tissue pressure (198).

Extracellular matrix binding receptors also are key players in tumor progression. Collagen I is the most abundant and well characterized component of interstitial matrix in PDAC. Collagen binds to integrins or DDR1 located on PDAC cells, inducing important downstream signaling pathways. Binding of collagen I to integrin on PDAC cells promotes proliferation, migration and inhibits apoptosis of tumor cells through an autocrine loop (199). Collagen I-Integrin signaling also promotes migration of PANC-1 and UlaPaCa cells through activation of FAK (200). FAK activation by this complex could lead to disruption of E-cadherin, promote Wnt activation and thereby regulate EMT (201, 202). The binding of collagen I to DDR1 activates FAK-related protein tyrosine kinase (PYK2), resulting in the expression of the EMT marker N-cadherin (203). Furthermore, binding of collagen I to DDR1 together with transmembrane-4-L-sox-family member 1 (TM4SF1) promotes invadosome formation, induces cell migration and promotes MMP-2 and -9 expressions (204, 205). Another study showed that high levels of palladin expression in PCSs enhance their ability to remodel the ECM by regulating the activity of Cdc42, which promotes invadosome formation as dots or rosettes in PSCs and tumor cell invasion (206). However, it has been reported in PDAC that PSCs can regulate matrix degradation by the activity of the large GTPase Dynamin 2 promoting tumor invasion, independent of invadopodia formation (207). Indeed, PSCs are able to promote tumor cell invasion by degradation of the matrix, dependent or independent of invadosome formation.

Finally, PSCs can directly interact with cancer cells, promote local tumor growth, and co-migrate with cancer cells to distant metastatic sites, establishing stromal abundant tumors beyond the pancreas. Additionally, activated PSCs and cancer cells produce pro-angiogenic factors, which promote neo- angiogenesis and support cancer cell growth and survival under a hypoxic tumor-microenvironment (208, 209).

To conclude, ECM in PDAC is one of the hallmarks of cancer and promotes PDAC progression. Little is known about the protective role of ECM in PDAC and needs further investigation. We could hypothesize that the aggressiveness of this cancer could be due to the absence of a protective role of the ECM or stromal cells compared to other cancers.

## Colorectal Cancer

### ECM Composition and Function

**FIGURE 4:**
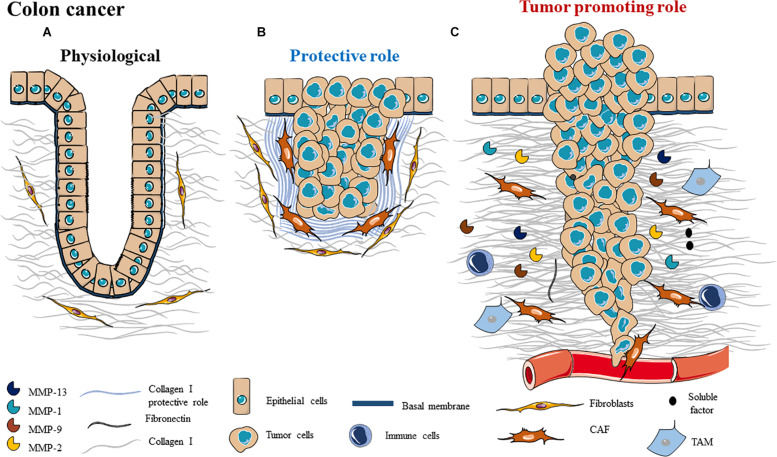
Schematic representation of ECM composition **(A)** and ECM dual role as a **(B)** protective barrier or as a **(C)** tumor promoting role in colon cancer.

In colon, in physiological conditions, colonic epithelial cells are anchored to the BM and act as a physical barrier with absorptive and exocrine functions (Figure 4A). BM is synthetized and secreted by epithelial and mesenchymal cells and separates the colon mucosa from its submucosa (210, 211). BM is composed of collagen IV, proteoglycan perlecan and glycoproteins such as laminin, fibronectin and nidogen (212). Stromal ECM is composed of similar components, but collagen IV is substituted by collagen I produced by resident fibroblasts.

Colorectal cancer (CRC) is the third most common cancer worldwide. An orderly ECM confers unique biomechanical properties in order to assure the regulation of cell proliferation and tissue homeostasis. During cancer, after BM degradation, abnormal ECM deposition and stiffness are observed, which correspond to desmoplastic reaction, promoting tumor progression (12).

Desmoplasia defines the abundant collagenous stroma surrounding parenchymal cells that is deposited after BM degradation. Fibroblasts are activated into myofibroblasts and become the primary producers of ECM in response to desmoplasia, leading to dramatic tissue remodeling (213). ECM of the CRC desmoplastic reaction is composed by collagen types I, III, IV, and V, proteoglycans (biglycan, fibromodulin, perlecan and versican) and small leucine-rich proteoglycans (SLRPs) decorin (214). Desmoplastic reaction prognosis is controverted in colorectal cancer: some studies report that it has a pro-tumor role but most of the studies describe a protective role, which is associated to good prognosis (214, 215) (Figure 4B). Therefore, it is important to study which ECM elements involved in the desmoplastic reaction are protectors or promoters of tumor progression.

### ECM Evolution During Cancer

#### Protective Role

In a study from 2011, Coulson-Thomas et al., showed that co-culture systems with colorectal cancer cell lines and fibroblasts promote an increase in ECM density which could inhibit the migration and invasion of CRC tumors. The desmoplastic collagen fibers were thicker than in normal tissue and arranged into parallel bundles with an altered orientation. This study demonstrated a protective role of CRC desmoplastic reaction by forming a barrier which can restrain tumor growth by creating an increased pressure, preventing tumor invasion of the surrounding tissue (214) (Figure 4B). A clinical study showed that desmoplasia is a protective factor for survival in patients with CRC. Thus, desmoplasia could prevent cancer cell invasion by building a barrier around the tumor (215).

However, for now, no study analyzes how and how long the protective barrier of desmoplasia needs to become pro-invasive and requires investigating. It could be due to collagen up-regulation as well as other ECM components such as fibromodulin, biglycan and fibronectin surrounding CRC. We could hypothesize that these components could first act as a protective barrier around the tumor cells; the pressure and stiffness then become too high in tumor cells which continue to proliferate which leads to the disruption of the protective barrier, allowing invasion and migration of tumor cells.

#### Tumor Promoting Role

Basement membrane disruption participates in tumor progression by releasing angiogenic, growth stimuli and chemotactic factors in order to promote tumor angiogenesis, growth and cell proliferation. For example, laminin 332 degradation promotes EGFR activation, causing a decrease of cell matrix adhesion enhancing migration (216). In CRC, loss of BM integrity is correlated to metastatic potential.

During cancer, the newly deposited collagen I replaces the proteolytically degraded ECM proteins by secreted proteases. This change can cause cellular migration which is predominantly oriented along radially aligned collagen fibers, promoting invasion. In physiological conditions, collagen fibers are disposed in the epithelium stroma with an angle of 10°, whereas in CRC, collagen fibers are thicker and present an angle of 50° (217). Furthermore, it has been demonstrated that ordered collagen fibers and an increase in collagen density are associated with CRC, demonstrating the main role played by collagen in malignant tissue transformation (218) (Figure 4C). In CRC, ECM elasticity ranges from soft and compliant to stiff and rigid. As mentioned before, tissue stiffness can be increased by enzymes such as LOX, which can crosslink collagen. In CRC cells, LOX is upregulated leading to increased tissue stiffness and activation of Src/FAK pathway promoting proliferation, invasion and metastasis (219, 220). Furthermore, at clinical level, LOX upregulation is associated with poor prognosis of CRC (221). Crosslinked collagen activates YAP and TAZ, promoting malignancy of CRCs.

A recent study analyzes the changes of the ECM at different stages of CRC and their effect on proliferation of cancer cells. It was shown that expression of MMP-2 and type I collagen are positively correlated to the stages in CRC. Collagen I expression is the highest in stage III and stage IV and lowest in normal tissue and stage I. The expression of MMP-9 is also higher in CRC, mainly in stage III. As regards collagen IV and TIMP-3, their expression is inversely correlated to CRC stages (221).

The binding of ECM elements to ECM receptors promotes tumorigenesis. Binding of collagen I to DDR1 promotes local invasion of primary CRC cells and promotes their dissemination. DDR1 overexpression is associated with poor prognosis in CRC patients (222). Binding of collagen I to DDR2 promotes cell proliferation, migration, invasion and peritoneal dissemination of colon cancer cells (223). Binding of collagen I to α2β1 integrin activates the pro-survival PI3K/AKT signaling pathway; resulting in the tumor promotion in CRC cells. This complex allows activation of transcription factor SNAIL; which in turn downregulates the expression of E-cadherin, inducing EMT and distant metastasis (224). Overexpression of CD44 is associated with poor prognosis of CRCs. The binding of HA to CD44v6 improves cancer cell proliferation, invasion, metastasis and resistance in colon cancer. The binding of osteopontin to CD44v6 also improves proliferation, invasion and metastasis of CRC cells (225). CAFs also improve the adhesion and migration of CRC through upregulation of CD44 in cancer cells (226). One study demonstrated that CD44 expression in CAFs maintains stem-cell properties of CRC cells but the exact molecular mechanism is not known. Furthermore, CD44 expressed by CAFs may interact with CRC cells to support cancer cell survival in hypovascular areas but it needs further investigations (54).

Besides collagen I, other proteins are deregulated in ECM of CRC. A downregulation of proteins such as keratin or collagen IV has been found in CRC tissues compared to normal tissues (227). During tumor invasion and metastasis, tumor cells directly secrete degradative enzymes and induce CAFs, inflammatory cells and the endothelial cells to produce proteolytic enzymes to degrade ECM. In CRC, MMP-1 and MMP-13 collagenases and MMP-2 and MMP-9 (two gelatinases) expression correlates to advanced CRC stage and poor prognosis (228). Different co-culture of CRC cell lines and TAM cell lines cause the upregulation of tumor cell-derived MMP-2 and MMP-9 expression and secretion, with increased tumor invasiveness and migration (229). Proteases such as ADAM9, ADAM10, TSLI and MMP-1, -2, -9, -11, and -12 have been found in colon primary tumor but not in metastasis, suggesting their role in migration of primary tumor cells (230). Myofibroblasts also promote CRC invasion by secreting soluble factors such as HGF and SPARC or by remodeling the ECM (231, 232). Myofibroblasts may interact directly with tumor cells by leading collective tumor cell invasion, through a process dependent on the Rho-GTPase effector ROCK (233).

Colorectal cancer cells are able to form invadosomes organized in dots in order to invade (234–236). Invadopodia formation could be mediated through activation of ROCK-II, modulating MMP-2 and -13 expressions and activities and by Smad 4-independent BMP signaling in CRC cells. Src activation could also induce Nox A1 phosphorylation, this will; in turn; lead to reactive oxygen species (ROS) generation promoting invadopodia formation (235–237). However, no study analyzes if these cancer cells are able to form linear invadosomes when they are seeded on collagen I. As expected, proteases were peculiar of primary colon tumor: ADAM 9, 10, TSL1 and MMP1, 2, 9, 11, and 12 have been found solely in colon tumor (230) and not in the metastasis, suggesting their role in the migration process. In another paper, the paired biopsies from tumor and its normal counterpart were obtained from 13 patients. Fifty-six proteins have been identified in the insoluble tissue fraction, after the extraction of lipids and soluble proteins. The digested peptides from ECM fraction were analyzed using a nano-ESI source by means of label-free quantitation approach (e.g., solely based on measurements of observed peptide ion peak intensities). The obtained data for Beside collagens, other ECM proteins are deregulated in CRC. One study report that MAGP2 (Microfibrial-associated glycoprotein 2), which is ECM component, is upregulated in CRC tissues compared to adjacent tissue, promoting proliferation, migration and invasion of cancer cells and the increase in it promoted malignant phenotypes of CRC cells including proliferation, migration, and invasion. Microfibrial-associated glycoprotein 2 can increase expression of the downstream genes of Notch, including HES1, Slug, Snail, matrix metalloproteinase (MMP) 2, MMP9, whereas its decrease Kruppel-like factor 4 (KLF4) expression. In this study they hypothesized that MAGP2 could be secreted by cancer cells or by CAF (238).

Furthermore, it has recently been shown that citrullined ECM proteins are characteristic of colon cell metastasis in the liver, suggesting that this process is important for the metastatic journey. Citrullination is the deamination of arginine residues to form peptides containing the non-coding amino acid citrulline. This process is a well-recognized characteristic of chronic inflammation, as demonstrates in autoimmunity where ECM proteins are extensively citrullinated. In CRC, citrullination is catalyzed by PAD4 which is produced by tumor cells, then PAD4 is delivered to the liver metastatic ECM by extracellular vesicles (239). ECM citrullination is a driver of human CRC liver metastasis.

To sum up, ECM of CRC evolves during tumor progression. The ECM first acts as a protective barrier to restrain tumor growth to local area and subsequently becoming a key player in tumor progression. Desmoplasia seems to act as a protective barrier and is a good prognostic in patient with colon cancer. It seems that the same element in colon ECM could have both a protective or a tumor promoting role. However, it would be interesting to study how the microenvironment dynamic influences this switch from protective to tumor promoting role.

## Melanoma

### ECM Composition and Function

**FIGURE 5:**
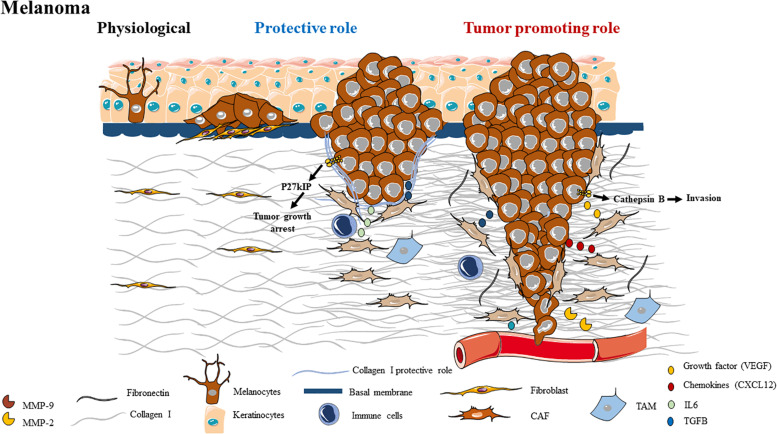
Schematic representation of ECM composition and ECM dual role as a protective barrier or as a tumor promoting role in melanoma.

Mammalian skin is composed of a multi-layered epithelium (Figure 5). The outer surface of the skin, the epidermis, consists of a keratinized stratified squamous epithelium. The epithelium rests on a layer of nourishing fibroelastic connective tissue called the dermis, which mainly consists of type I collagen. The dermis is connected to the underlying tissue by a layer of loose connective tissue, the hypodermis or subcutaneous layer, which contains varying amounts of fat tissue. Skin is composed of cells such as fibroblasts, endothelial cells, keratinocytes and ECM (240).

The separation between the epidermis and the dermis is a BM (Figure 5). In skin, BMs are composed of laminin, type IV collagen, nidogen, and perlecan, a heparan sulfate proteoglycan (241). In contrast, the tensile strength and elasticity in the dermis underneath is determined by ECM, composed of collagen types I (80%), III (15%), and type V (5%), microfibrils, elastic fibers, proteoglycans, glycosaminoglycans and water (242). In normal dermis, collagen fibers exhibit a random, “basket-weave” structure (243). The cutaneous ECM is constantly remodeled throughout the lifespan, for example during wound-healing or aging (243).

Melanoma is a cancer that arises from melanocytes. This very aggressive skin cancer develops in very rich in fibrillar type I collagen environment (Figure 5). In physiological conditions, keratinocytes modulate behavior of melanocyte population and the dermally located fibroblasts synthesize the ECM. During the initiation of melanomagenesis, melanocytes accumulate sufficient mutations to degenerate, notably through the aberrant activation of an oncogene such as the *BRAF V600E* mutation. Melanoma cells, therefore, hyper-proliferate on the surface of the skin during radial growth. Subsequently, the cells deeply invade the deep layers of the skin, after having degraded the BM separating the epidermis from the dermis. The transition from radial to vertical growth phase in melanoma is associated with loss of E-cadherin expression, increased N-cadherin expression and increased expression of αvβ3 integrin, leading to secretion of the antiapoptotic factor bcl-2 and MMP-2, an endopeptidase that degrades collagen IV at the BM (244–246). Additionally, the shift from E-cadherin to N-cadherin expression allows melanoma cells to interact with fibroblasts and vascular endothelial cells to better facilitate migration and intravasation (247). The fibroblasts also become activated, resulting in increased growth factor production leading to a hyperproliferative microenvironment that supports growth of many cell types and collagen I synthesis (248). Finally, the tumor is fully competent to invade and metastasize to distant organs. Once metastasis to distant organs has occurred, the tumor enters its final stage and is termed metastatic melanoma.

### ECM Evolution During Cancer

#### Protective Role

The ECM can first act as a protective barrier against melanoma progression (Figure 5). In the dermis, the major component of the extracellular matrix is type I collagen, which is synthesized mainly by fibroblasts. It has been established that collagen I acts as a protective barrier in tumor progression and proliferation (249). In the same study, they showed that contact with fibrillar collagen inhibits the proliferation of malignant and highly metastatic M24met cells. Inhibition of proliferation is due to the binding of collagen to α2β1 integrin which induces an increase in p27KIP1 mRNA and protein, promoting growth arrest in the G1/S transition and inhibition of cyclin E-associated kinase activity (249).

During the early phase of melanomagenesis such as Radial Growth Phase (RGP), co-culture of fibroblasts with RGP melanoma cells represses tumor growth; whereas advanced melanoma cells acquire an ability to escape such control mechanisms (250). It is possible that dermal fibroblasts form a physical barrier that blocks melanoma cells to migrate and invade the surrounding tissues. Another hypothesis, regarding the inhibitory effect of dermal fibroblasts, is that dermal fibroblasts could recruit immune cells by secreting interleukin-6 (IL-6) (251).

#### Tumor Promoting Role

Cancer-associated fibroblasts at the level of the primary tumor are called melanoma associated fibroblast (MAFs) and are involved in melanoma progression (Figure 5). Fibroblasts can be activated by chemical factors secreted by melanoma cells, inducing fibroblasts to migrate toward, surround, and then infiltrate the tumor mass. For example, in melanoma, the secretion of TGF-β by tumor cells allows the activation of MAFs (252), which are able to synthesize and deposit ECM proteins such as collagen, fibronectin and tenascin (253, 254).

PDGF and bFGF could increase the production of glycosaminoglycan (GAG) from MAFs (255). Oxidative stress induced by hypoxia in the melanoma as well as factors secreted by melanoma cells stimulate MAFs to secrete cytokines and growth factors such as VEGF, stromal derivative factor-1 (SDF-1 or CXCL12) and IL-6 thus promoting invasion into the melanoma (256, 257).

Melanoma associated fibroblast are also able to remodel the ECM by MMP-1, MMP-2, MMP-13, and MT1-MMP (MMP-14) secretion, which could influence the motility and invasiveness of melanoma cells (205, 258–261). In primary and metastatic melanoma, it has been shown that up-regulation of FAP-α expression (an active serine protease which could degrade type I collagen) enhances ECM remodeling, tumor cell growth and migration (262, 263).

Collagen I receptors are also involved in tumor progression. CD44 expression is associated with poor prognosis of melanoma, and different studies have shown that binding of collagen I or HA to CD44 promote tumor progression (264, 265). The binding of collagen I to α2β1 integrin promoting cathepsin B-mediated invasiveness was associated with secreted acidic and cysteine-rich proteins in melanoma (266). The binding of collagen I to DDR1 enhances invasion and the binding of collagen I to DDR2 induces MMP-2 and MMP-9 expressions as well as Erk/NF-κB signaling pathways to promote invasion (267). Despite the abundance of collagen I, melanoma progression is characterized by the increase of other matrix proteins such as tenascin-C and fibronectin. These two proteins could affect the organization of collagen fibers. It has been shown that MAFs facilitate tumor invasion through αVβ3 integrin-dependent fibronectin secretion, which induces mechanical changes in the ECM through the contraction of collagen fibers (268). It has been previously demonstrated that biglycan expression is involved in matrix contraction and increased in matrix stiffness which induce β1 integrin expression, promoting invasion of melanoma cells (269). However, most of the studies focus on ECM stiffness and its protective role during resistance to the treatment. It has been shown that an increase in ECM stiffness upon exposure of BRAF inhibitor promotes a protective matrix environment during resistance to treatment (270, 271). This increasing stiffness leads to the re-organization of β1 integrin into focal adhesions and elevated pFAK levels (271). The binding of fibronectin to α4β1or αVβ3 integrins promote melanoma cell invasion (268, 272).

Tks4 and Tks5 adaptor proteins are key players in melanoma growth and metastasis *in vitro* and *in vivo*, promoting invadopodia formation by MT1-MMP regulation (273). Another study showed that, invadopodia formation in melanoma cells could be regulated by crosstalk between receptor tyrosine kinases AXL and ERBB3 (274). Our data report that when melanoma cells are seeded on collagen I matrix, there is invadosome reorganization into linear invadosomes (51).

Finally, MAFs expresses a lot of proteins which are key players for melanoma cell metastasis. MAFs secrete tenascin C and periostin, which are required for the development of a CSC phenotype and the formation of metastatic sites. MAFs are also able to secrete the matricellular protein CCN2, which is required for melanoma metastasis (275, 276). Furthermore, different crosstalk between MAFs and melanoma cells are involved in metastasis of melanoma cells. For example, CXCR4 (CXC chemokine receptor-4) is expressed on the surface of melanoma cells, while its ligand CXCL12 is released by MAFs in the tumor microenvironment, promoting the migration and metastasis of melanoma cells to distal metastatic sites through interaction with CXCR4 expressed on tumor cells (277–279). Besides, HGF secreted by MAFs induce fibronectin expression and associated matrix assembly, which promotes melanoma cell metastasis (280).

Extracellular matrix in melanoma firstly acts as a protective barrier to avoid tumor progression. Then, ECM becomes an essential partner in order to facilitate migration, invasion, metastasis and resistance in the melanoma. It could be important to study the crosstalk between cancer cells and stromal cell in the promotion of ECM remodeling, degradation and invasion, in a physiological matrix model, in different skin acellular models that exist (262, 281).

## Similarities and Differences Between the ECM of the Five Cancers

We note that these five cancers share similarities (Table 1). First, a crosstalk between cancer and stromal cells, where cancer cells could activate stromal cells into stromal cancer cells, promoting enhancement of ECM deposition. The stromal cancer cells in turn are able to secrete growth factors and cytokines to promote the invasion of tumor cells. Second, these 5 cancers also share some similarities in their ECM composition: after BM disruption, collagens I, III, and V, proteoglycans, glycosaminoglycans and elastic fibers accumulate. Whereas after BM disruption there generally is a decrease of collagen IV, we note that liver cancer ECM showed an upregulation of collagen IV. Furthermore, at late stages of tumor progression, biomechanical properties of the matrix, such as the alignment of ECM constituents have been correlated to cell invasion and poor prognosis. Moreover, a recent paper showed the importance of crosstalk between stromal cells and ECM to promote breast cancer cell migration (282). Indeed, CAFs, through cell collision guidance, induce their own alignment, which in turn, promote ECM alignment. This increased ECM alignment promotes tumor cell invasion, suggesting that the cancer ECM anisotropy is a key characteristic to take into consideration while studying cancer.

**TABLE 1:** Similarities and differences in composition and crosslinking of the tumor associated protective or tumor promoting ECM in each cancers.

Stroma composition				
	Protective	Pro-tumoral		
	Composition	Composition	Signaling pathways	Cross-linking
Breast cancer	– Collagens I, III, V, fibronectin, laminin, decorin – Myoepithelial cells – CAFs	– Collagens I, III, V – Fibronectin, fibrin, hyaluronan, versican, osteopontin, tenascin, periostin	CD44 → PDGFRβ/Stat3 CD44 → MAPK or AKT → Invasion, migration, survival Collagen I → Integrin α11 → PDGFRβ/JNK → Invasion → DDR1 → Invadosomes formation → DDR1 + IGFRI → Proliferation and migration Collagen IV → DDR1 → NFκB → Resistance Fibronectin → Erk or Stat 3 → EMT Laminin → Integrin → Invasion and migration	LOX LOXL2 LOXL3 LOXL4
Liver cancer	– Collagens I, III – CAFs	– Collagens I, III, IV – Tenascin, osteopontin, laminin, chondroitin sulfate	Collagen I → β1 integrin → MAPK → prosurvival signal CD44 → MDM2 nuclear translocation → Akt → tumor progression TGFB → collagen I → DDR1 → LOXL2 → Invadopodia Collagen I → DDR1 → Erk → SNAIL → EMT → DDR2 → ERK → MMPs → Invasion	LOXL2
Pancreas cancer	– Byglican, lumican: associated to better prognosis	– Collagens I, III – Fibronectin, vitronectin, glypican, SPOCK1, HA	Hyaluronan → CD44 → survival, proliferation, invasion Collagen I → Integrin → FAK → migration → DDR1 → PYK2 → EMT → DDR1/TM4SF1 → invadosome formation	LOX TG2
Colon cancer	– Collagens I, III, IV, V, biglycan, fibromodulin, perlecan, versican, decorin	– Collagens I, III, V – Byglican, perlecan, versican, fibromodulin, biglycan, fibronectin	Collagen I → DDR1 → Invasion → DDR2 → Proliferation, migration, invasion → α2β1 → PI3/Akt → tumor progression → α2β1 → Snail → EMT HA → CD44v6 → proliferation, invasion, resistance Osteopontin	LOX
Melanoma	– Collagen I – Fibroblast	– Collagen I – Tenascin-C, fibronectin, periostin, osteopontin, SPARC, CCN3	Collagen I → α2β1 → Cathepsin B → invasion → DDR1 → Invasion → DDR2 → ERK/NFκB → Invasion	Fibronectin Biglycan Tenascin C

Despite similarities in these different types of matrix, we also note major differences (Table 1). For instance, collagen crosslinking is mediated by LOX only in breast and colon cancers, whereas in pancreas, crosslinking could be also mediated by transglutaminase 2 (30, 32, 81, 82, 184, 221). In liver cancer, collagen crosslinking is mediated by LOXL2 only. Regarding melanoma, tenascin C and fibronectin affect the organization of collagen fibers and biglycan is involved in matrix contraction and increased matrix stiffness (175, 268). To our knowledge, no study reports the role of LOX in collagen crosslinking in melanoma.

One other major difference is that TAEM in breast, liver, colon and melanoma cancer has an anti-tumor role to restrict tumor growth at the primary site, whereas this is not observed in pancreatic cancer. ECM in breast cancer is the most studied and described. One of the specificities of breast cancer is that myoepithelial cells act as a protective barrier around the tumor cells and are able to decrease the secretion of MMP-2, MMP-9, and MT1-MMP (94–96). In addition, they can secrete protease inhibitors or angiogenic inhibitors, several tumor suppressors in order to prevent tumor growth, invasion and metastasis (81, 82, 84). CAFs are often associated with poor prognosis in cancer, whereas in liver and in breast cancer, CAFs can also participate to the protective role of ECM. In breast cancer, CAFs have been shown to secrete factors which are associated with decrease metastasis (87–89). ECM of liver cancer, colon cancer and melanoma present some similarities with regards to the protective effect. Indeed, at the early stages of tumor progression, they all show a structure like a capsule made of collagen and fibroblasts around the tumor in order to restrict tumor growth (214, 215). No study analyzes how the protective barrier of desmoplasia becomes pro-invasive, which requires further investigations. We could hypothesize that collagen secretion could first act as a physical protective barrier around the tumor cells. Then, the pressure becomes too high by tumor cells which continue to proliferate, that the protective barrier is disrupted, allowing invasion and migration of tumor cells. We could also postulate that when fibroblasts are activated into CAFs, there is an upregulation of ECM component secretion promoting pressure around the tumor, leading to the disruption of the protective barrier and then to cancer progression. Regarding pancreatic cancer, one of the most aggressive cancer, there is no collagen or fibroblast protective barrier at early stages. Thus, maybe the aggressiveness of this cancer at beginning stages could be due to the lack of the protective barrier. In all of the cancers discussed above, we could not find any study that analyzes the transition between protection and this pro-invasive effect. New epigenetic mutations in cancer cells that promote proliferation and invasion of the protective barrier - immunity or metabolic stress - could be at the origin of this transition. It would be crucial to study the elements which could induce this switch, in order to promote protective role of ECM in cancer and restrain tumor growth.

To sum up, the complexity and heterogeneity of each tumor matrix is due to the architecture and organization of each organ. In addition of this inter-tumor heterogeneity, matrix heterogeneity can also be observed at the level of the same tumor and each tumor structure could have a specific matrix.

## Discussion

In the last decade, the role of ECM in cancer has been widely studied and gained more and more importance. During cancer progression, ECM is constantly remodeled, and is the result of a balance between secretion and degradation. ECM evolves constantly from primary tumor to metastasis site including pre-metastatic niche. Therefore, there is modification of ECM composition and organization in the pre-metastatic niche for cancer cell to become dormant or to grow and form metastasis.

The crosstalk between tumor and stromal cells controls this balance. Tumor evolution leads to TAEM creation, which is essential in the tumor progression. In order to interact with TAEM, stromal and cancer cells need to express ECM receptors including collagen receptors promoting malignant phenotype of tumor cells such as invasion, migration and proliferation.

However, this scheme of ECM involvement in cancer progression is too simple and need to be adapted to each organ, cancer and cancer stages. We showed, in this review, that each cancer has its own matrix, with its own composition, its own molecules promoting crosslinking, therefore they present specific pro-tumor or protective effect.

The ECM is well-known and well-studied for its tumor promoting role. However, it is very important to note that, at the beginning of a large number of cancers, ECM first could serve as a protective barrier. It could be complicated to develop therapies against ECM due to its heterogeneity as well as its dual role as a pro or anti-tumor. However, there is a real need to understand the dynamics of the microenvironment, in order to determine when and how the protective barrier could became pro-tumor. This could allow development of a therapeutic strategy to enhance protective role of the ECM and control the disease by preventing or delaying the pro-tumor role of the ECM.

## Author Contributions

MS and MR prepared the figures and wrote and edited the manuscript. FS edited the manuscript. All authors contributed to the article and approved the submitted version.

## Conflict of Interest

The authors declare that the research was conducted in the absence of any commercial or financial relationships that could be construed as a potential conflict of interest.
